# RadPulse: A Free, National, Near-Peer Online Prescribing Safety Assessment Preparation Course for United Kingdom Final-Year Medical Students

**DOI:** 10.7759/cureus.116029

**Published:** 2026-09-10

**Authors:** Muhammad Haris Irfan, Mohammed Al-Bajari, Henilkumar R Patel, Syed Haider Ali Bukhari

**Affiliations:** 1 Postgraduate Department, Manchester University NHS Foundation Trust, Manchester, GBR; 2 Internal Medicine Department, Royal Bolton Hospital, Bolton, GBR; 3 General Surgery Department, Royal Blackburn Teaching Hospital, Blackburn, GBR

**Keywords:** kirkpatrick evaluation, medication safety, near-peer teaching, online learning, prescribing education, prescribing safety assessment, undergraduate medical education

## Abstract

Introduction: Prescribing errors are a common, costly source of preventable harm in the UK National Health Service, and preparation for the mandatory prescribing safety assessment (PSA) is widely reported to vary between medical schools. RadPulse was a free, national, near-peer online PSA course delivered by junior doctors to UK final-year medical students.

Methods: Nine live 90-minute sessions were delivered via Microsoft Teams (Microsoft Corporation, Redmond, WA) between October 2024 and January 2025, covering all eight PSA domains through timed exam-style questions, worked clinical reasoning, and live navigation of the British National Formulary and Medicines Complete. Data were collected via a registration form, session feedback forms, and a voluntary postexamination outcomes form. Likert responses were scored 1-5; a Wilson 95% confidence interval (CI) was calculated for the reported pass proportion, and exploratory, uncorrected Spearman correlations examined score-attendance and school-level provision-uptake relationships.

Results: A total of 515 unique students registered from 13 UK medical schools. At registration, mean self-rated PSA preparedness was 2.09/5 (standard deviation (SD), 0.86), and 272/515 (52.8%) rated their medical school's PSA provision 1 or 2 out of 5. Across 1,046 session feedback responses, 98.9% agreed content was appropriately pitched (mean 4.80/5). Self-rated preparedness rose to 3.87/5 (SD, 0.64) by Session 8 among an anonymous, unlinked sample. Of 98 students (19.0% of registrants) who completed the outcomes form, 97 (99.0%; Wilson 95% CI, 94.4-99.8) reported passing the PSA, compared with a national first-attempt benchmark of 91%-97% for cohorts through 2023. Score was not associated with attendance (Spearman rho = 0.07, p = 0.55).

Conclusions: A free, near-peer, online prescribing course delivered by junior doctors achieved national cross-institutional reach and high learner satisfaction, with a high self-reported pass rate among students who provided an outcome. Self-selection, unlinked data, an unvalidated preparedness measure, and a 19.0% outcomes response rate limit causal interpretation; the pass figure is an optimistic upper bound rather than a population estimate. The approach may offer a low-cost model that other institutions could consider replicating.

## Introduction

Prescribing is the clinical task performed most frequently by newly qualified doctors and the one in which errors are most consistently documented. The EQUIP study found errors in 8.4% of medication orders written by foundation year (FY)1 doctors, a higher rate than any other grade, including consultants, with patient harm in a significant proportion of cases [1]. Elliott et al. estimated the direct National Health Service (NHS) cost of avoidable medication errors at £98.5 million a year [2], and a more recent modeling study put the total at 1.8 million transition-of-care errors annually, contributing to over 40 preventable deaths and 36,500 excess bed-days [3].

The Prescribing Safety Assessment (PSA) was developed jointly by the British Pharmacological Society and the Medical Schools Council as a two-hour, eight-domain online examination covering prescription writing, prescription review, planning management, communicating information, calculation skills, adverse drug reactions, drug monitoring, and data interpretation. Piloted in 2012-2013 and rolled out to all UK medical schools from 2014, the PSA has been mandatory for progression from FY1 to FY2 since August 2016 and now sits within the General Medical Council's graduate outcomes framework [4]. Published national first-attempt pass rates have ranged from 91% to 97% across successive cohorts to 2023 [5,6].

Many students nonetheless report feeling underprepared for the PSA and attribute this in part to their own medical school's teaching [7]. Over half of RadPulse registrants rated their own medical school's PSA provision 1 or 2 out of 5, indicating a widely perceived gap in institutional teaching. Near-peer teaching, in which instruction is delivered by individuals only slightly senior to the learner, may be well suited to this gap. Cognitive and social congruence describes how near-peer tutors teach to the exam's real demands and pitch at the right level, having faced the same difficulties recently [8]. A systematic review of 22 near-peer surgical teaching studies found outcomes at Kirkpatrick Levels 1 and 2 to be at least equivalent to those of faculty instruction [9], and a recent single-center study reported improved PSA scores following pharmacist-led peer teaching [10]. Whether such teaching can be implemented across institutions with minimal infrastructure, which components are essential, and whether any benefit extends beyond confidence and examination preparation are less clear.

Online delivery considerably extends the reach of near-peer teaching. A virtual near-peer objective structured clinical examination program across 13 UK universities reported improved confidence in 90%-96% of participants [11], and a comparable free, near-peer, open-access surgical education model reached over 10,000 attendances at essentially no cost [12].

Whether near-peer PSA teaching can be delivered at national scale with minimal infrastructure, and what its reach and acceptability look like when it is, remains largely unexamined outside single-institution settings. RadPulse was established to address this gap. A free online course was delivered by doctors who had sat the PSA themselves in the preceding two years, and was open to any UK final-year student who wished to join. We report its national reach, learner satisfaction, self-rated preparedness, and PSA outcomes after its first year, and set out the further evidence needed before any claim of improved prescribing performance could be made.

## Materials and methods

Program design

RadPulse ran nine free live sessions via Microsoft Teams (Microsoft Corporation, Redmond, WA) between October 2024 and January 2025, finishing shortly before the January 29, 2025, national PSA sitting. Each session followed a consistent 90-minute format: exam-style questions followed by a detailed walkthrough of each answer, including why incorrect options were wrong and how to locate relevant information in the British National Formulary (BNF) and Medicines Complete under exam conditions. Sessions covered all eight PSA domains. Table 1 lists the full nine-session curriculum, and Figure 1 maps each session to its corresponding PSA domain and representative topics. Teaching was delivered by junior doctors who had sat the PSA within the preceding two years; content was not independently reviewed by a senior clinician or pharmacist beyond the tutors' recent examination experience. The course was promoted through social media and peer networks with no institutional support or external funding.

**Table 1:** RadPulse session curriculum PSA: Prescribing Safety Assessment; N/A: not available

Session	Title	PSA domain 1	PSA domain 2
1	Introduction to the PSA, top tips, example format questions	Prescription writing	N/A
2	Prescribing, part 1	Prescription writing (continued)	N/A
3	Prescribing, part 2	Prescription writing (continued)	N/A
4	Prescription review and planning management	Prescription review	Planning management
5	Providing information and calculation skills	Communicating information	Calculation skills
6	Adverse drug reactions and drug monitoring	Adverse drug reactions	Drug monitoring
7	Data interpretation	Data interpretation	N/A
8	Question-and-answer session	All eight PSA domains	N/A
9	Mock examination	All eight PSA domains	N/A

**Figure 1:**
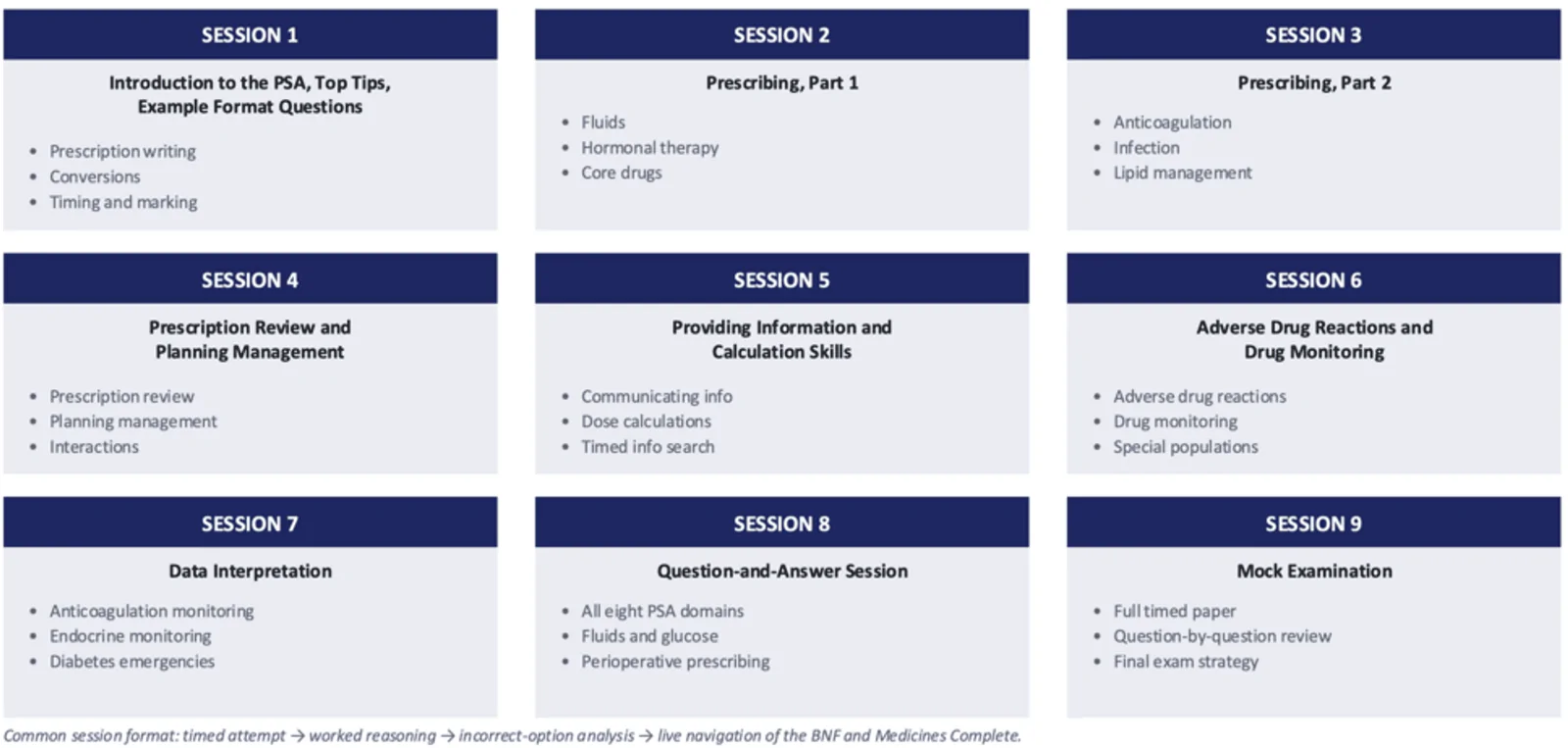
Nine-session curriculum mapped to PSA domains PSA: Prescribing Safety Assessment; BNF: British National Formulary Image credit: This is an original image created by the authors using Microsoft PowerPoint (Microsoft Corporation, Redmond, WA)

The design was intended to draw on worked-example, retrieval-practice, and distributed-practice principles from cognitive and educational psychology [13-15]: sessions were spaced across four months, and each question was followed immediately by a worked explanation; fidelity to these principles was not formally assessed.

Participants

All UK final-year medical students were eligible to register; participation and attendance were voluntary and free. The PSA is taken by final-year students at all UK medical schools, and 'final year' corresponds to year 4 at graduate-entry schools, year 5 at standard five-year schools, and year 6 where an intercalated year has been completed.

Evaluation instruments

Evaluation was structured around Kirkpatrick's four levels [16]: Level 1 (Reaction), satisfaction with content and delivery; Level 2 (Learning), self-rated preparedness and confidence, measured using an unvalidated single-item scale; Level 3 (Behavior), not assessed, as participants had not yet begun independent prescribing at follow-up; and Level 4 (Results), operationalized here as PSA examination outcome rather than the organizational or patient-level impact more commonly denoted by this level.

Three instruments collected data (see Appendixes A-C). A registration form captured medical school, year of study, self-rated PSA preparedness (1-5, where 1 = not at all prepared), and satisfaction with medical school PSA provision (1-5). Session feedback forms used five-point Likert items (strongly agree to strongly disagree) on content appropriateness and clarity of aims; Sessions 1-6 also asked about structure and flow, and Sessions 8 and 9 added numeric items on postcourse preparedness, confidence improvement, and overall usefulness (each 1-5), plus open-text fields on what was useful and what could improve. A voluntary post-PSA outcomes form, completed after the 29 January 2025 examination, recorded sessions attended and pass/fail result; percentage score was optional. No linked or pseudonymized identifiers were used, a design choice intended to protect anonymity and maximize response at the cost of individual-level comparison.

Statistical analysis

All analyses were descriptive. Duplicate registrations were removed by e-mail address. Likert responses were scored 5-1 (strongly agree to strongly disagree) and treated as interval data for calculating means and standard deviations (SDs), as is common in this literature; the underlying data are ordinal. "Agree or strongly agree" was the positive category. The registration-Session 8 preparedness comparison used nonmatched, anonymous samples and is presented descriptively, with no inferential test applied. PSA pass rate is reported against the published national benchmark of 91%-97% [5,6], with a Wilson 95% confidence interval (CI) calculated for the observed proportion. Spearman correlations explored complete-case score-attendance and school-level provision-uptake associations; both were exploratory and uncorrected for multiple comparisons, and should be read as hypothesis-generating. Open-text responses were coded inductively for recurring themes by two independent readers, with disagreements resolved by discussion; interrater agreement was not formally quantified. Analysis used Python 3.12 (Python Software Foundation, Wilmington, DE).

Ethical considerations

This project met Health Research Authority criteria for service evaluation and did not require NHS Research Ethics Committee approval. Voluntary survey completion constituted implicit consent to the use of anonymized data for educational research and publication. No patient data were collected.

## Results

Reach

A total of 521 registration submissions were received. After removing six duplicate email submissions, 515 unique registrants remained, all from 13 UK medical schools spanning Russell Group, post-1992, and independent institutions: Manchester (n = 136), Warwick (n = 80), Sheffield (n = 68), Plymouth/Peninsula (n = 58), Buckingham (n = 51), Lancaster (n = 40), Norwich/University of East Anglia (n = 28), Oxford (n = 19), Barts and the London/Queen Mary University of London (n = 16), University College London (n = 9), King's College London (n = 7), Hull York Medical School (n = 2), and St George's (n = 1). All were final-year students; the variation in numeric year reflects differences in program length across institutions.

Baseline preparedness and institutional provision

Mean self-rated PSA preparedness at registration was 2.09/5 (SD, 0.86, n = 515), skewed toward lower scores: 149 registrants (28.9%) rated themselves 1 out of 5 and 191 (37.1%) rated themselves 2 (66.0% combined), while only 17 (3.3%) rated themselves 4 or 5. Mean satisfaction with medical school PSA provision was 2.41/5 (SD, 1.00); 272 registrants (52.8%) rated it 1 or 2 out of 5.

Session feedback (Kirkpatrick Level 1)

A total of 1,046 feedback responses were collected across all nine sessions, ranging from 76 (Session 1) to 153 (Session 5). Table 2 presents the full session-by-session feedback breakdown. The mean score for "content pitched at an appropriate level" was 4.80/5 overall, with 98.9% of responses at 4 or 5. Aims were rated as clearly defined by at least 96.1% of respondents in every session, and no session fell below 94.7% positive on any item.

**Table 2:** Session-by-session feedback summary (Kirkpatrick level 1) Structure/flow item was not included in Sessions 7-9 SA: strongly agree; N/A: not available

Session	n	Content appropriate (% agree/SA)	Aims clear (% agree/SA)	Structure/flow (% agree/SA)	Mean (/5)
1	76	96.1	96.1	94.7	4.66
2	126	99.2	96.8	96.8	4.72
3	135	100.0	99.3	99.3	4.87
4	123	98.4	99.2	100.0	4.77
5	153	100.0	100.0	100.0	4.85
6	130	98.5	98.5	98.5	4.78
7	121	100.0	100.0	N/A (not asked)	4.83
8	83	100.0	98.8	N/A (not asked)	4.81
9	99	97.0	98.0	N/A (not asked)	4.83
All	1,046	98.9	≥96.1	≥94.7	4.80

Postcourse preparedness and confidence (Kirkpatrick Level 2)

By Session 8 (question-and-answer), mean self-rated preparedness was 3.87/5 (SD, 0.64, n = 83): 51 (61.4%) rated themselves 4 out of 5 and 11 (13.3%) rated themselves 5, compared with 3.3% rating themselves 4 or 5 at registration. Figure 2 summarizes this change alongside institutional provision ratings and Session 9 confidence and usefulness ratings. Registration and Session 8 samples were anonymous and unlinked, precluding a within-person comparison. At Session 9 (mock examination), 91.9% of respondents (n = 99) rated their confidence improvement 4 or 5 out of 5 (mean, 4.28; SD, 0.73), and 98.0% rated overall usefulness 4 or 5 (mean, 4.78; SD, 0.46).

**Figure 2:**
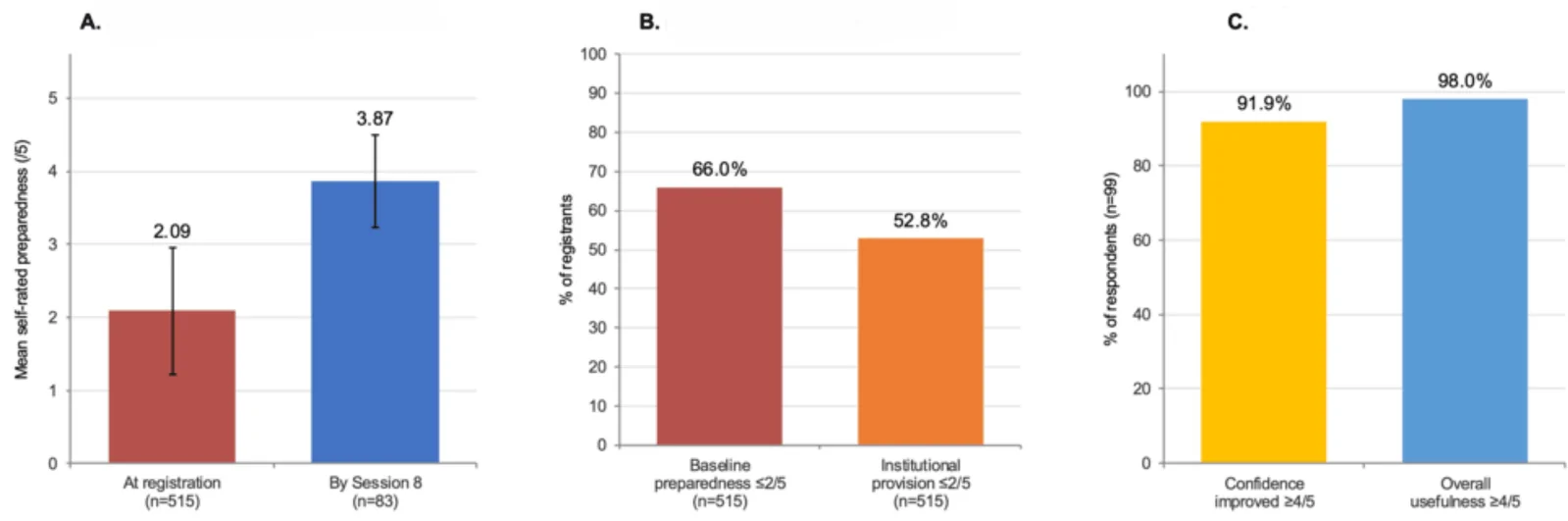
(A) Mean self-rated PSA preparedness with 95% CI at registration vs. by Session 8. (B) Share of registrants reporting baseline preparedness or institutional provision ≤2/5. (C) Session 9 ratings for confidence improvement and overall usefulness (n = 99) PSA: Prescribing Safety Assessment; CI: confidence interval Image credit: This image was created by the authors using Microsoft PowerPoint (Microsoft Corporation, Redmond, WA) from the study's own survey data

PSA examination outcomes (Kirkpatrick Level 4)

Ninety-eight students completed the post-PSA outcomes form (19.0% of 515 registrants), all giving a usable pass/fail answer, leaving 417 registrants with unknown outcomes. Of these, 97 (99.0%; Wilson 95% CI, 94.4-99.8) reported passing, against the national benchmark of 91%-97% [5,6], reflecting cohorts through 2023 and predating the January 2025 sitting attended by RadPulse students. The one nonpassing student had attended five sessions and scored 60%. Among the 97 passing students, 90 voluntarily disclosed their percentage (a further self-selected subset of an already self-selected sample), with a mean score of 86.7% (SD, 6.3; median, 86%; range, 70%-98%).

Three respondents gave their medical school as "Imperial" or "Imperail," a school not represented among the 515 registrants; whether these were RadPulse attendees registered under a different school name or a data-entry inconsistency could not be verified. These records were retained in the total rather than excluded.

Attendance and exploratory associations

Among the 98 outcome respondents, 26 (26.5%) attended all nine sessions, and all passed; 17 attended eight sessions (all passed); and 17 attended seven sessions (all passed). Median attendance was seven sessions (mode, 9; mean, 6.7).

Attendance was not associated with score among 80 complete pairs (Spearman rho = 0.07, p = 0.55), and school-level provision and uptake were directionally associated but not statistically significant (rho = 0.39, p = 0.19; 13 schools). Both correlations were exploratory and uncorrected for multiple comparisons.

Qualitative themes

Over 1,400 free-text responses across the registration and session feedback forms were coded for recurring themes. Four themes appeared consistently across all nine sessions; Table 3 presents these themes with representative participant quotes.

**Table 3:** Identified themes and representative participant quotes Quotes are drawn from anonymous free-text responses; no identifying information is included IV: intravenous; BNF: British National Formulary

Theme	Representative participant quotes
Worked questions with explanations of incorrect answers	“I really appreciate the explanations and reasoning for incorrect answers - for example, why IV furosemide is preferred over oral - and showing where the information can be found in the BNF.” (Session 3 respondent)
Live navigation of drug-information resources under exam conditions	“A lot of teaching programmes like to beat around the bush, but the explanations here included not only the science but how to use the BNF to find the answers.” (Session 9 respondent)
Clinical applicability (insulin, IV fluids, anticoagulation, antibiotics)	“The questions involved clinical knowledge and reasoning to figure out what medications were needed and what to look out for.” (Session 2 respondent)
Exam technique (domain structure, time allocation, high-yield facts)	“The calculation session was the most useful as I felt that to be very daunting; every session had practice questions which helped apply clinical knowledge into practice.” (Session 9 respondent)

Table 4 summarizes results across all Kirkpatrick levels assessed.

**Table 4:** Summary of outcomes across Kirkpatrick levels The 19.0% outcomes response rate means the pass figure is an upper bound, not a population estimate. The Wilson 95% CI gives the plausible range for the true pass rate given the sample size; the Spearman rho is a correlation coefficient ranging from -1 to 1 that indicates the strength and direction of an association between two ranked variables (0 = no association), and the accompanying p value indicates whether that association is likely to reflect chance, with p < 0.05 conventionally taken as statistically significant SD: standard deviation; PSA: Prescribing Safety Assessment; SA: strongly agree; CI: confidence interval

Metric	Value
Registration submissions received	521
Duplicate submissions removed	6
Unique registrants	515
UK medical schools represented	13
Baseline preparedness, mean (/5)	2.09
Baseline preparedness, SD	0.86
Baseline preparedness ≤2/5	66.0% (340/515)
Baseline preparedness ≥4/5	3.3% (17/515)
Institutional provision rating, mean (/5)	2.41
Institutional provision rating, SD	1.00
Institutional provision rating ≤2/5	52.8% (272/515)
Session feedback responses, total	1,046
Content pitched appropriately, mean (/5)	4.80
Content pitched appropriately, % agree/SA	98.9%
Postcourse preparedness, mean (/5), n = 83	3.87
Postcourse preparedness, SD	0.64
Postcourse preparedness ≥4/5	74.7% (62/83)
Session 9 confidence improved ≥4/5	91.9% (91/99)
Session 9 overall usefulness ≥4/5	98.0% (97/99)
Outcomes form submissions	98 (19.0% of registrants)
PSA pass rate	99.0% (97/98)
PSA pass rate, Wilson 95% CI	94.4%-99.8%
Mean PSA score disclosed (/100), n = 90	86.7
Mean PSA score disclosed, SD	6.3
Score-attendance correlation, Spearman rho	0.07
Score-attendance correlation, p value	0.55
School provision-uptake correlation, Spearman rho	0.39
School provision-uptake correlation, p value	0.19

## Discussion

RadPulse reached 515 registrants across 13 medical schools, recruited entirely through social media and peer networks without institutional support or funding. This spread across a broad and structurally diverse set of institutions suggests the perceived gap in PSA preparation was not confined to a small group of highly engaged students, although registration numbers alone cannot establish how representative or severe that gap actually was. The pattern fits a wider trend: free, socially distributed teaching of this kind has moved over the past decade from an informal, grassroots activity into a more structured and widely used part of clinical training, a trajectory documented in the free open-access medical education literature [17].

Qualitative feedback converged on explanations of incorrect answers, live demonstration of drug-information retrieval under exam conditions, and clinically framed rather than purely factual questions. These themes align with the near-peer teaching literature, in which cognitive and social congruence between tutor and learner is proposed as a mechanism of effectiveness [8], and they echo prior reports of encouraging outcomes from junior doctor- and pharmacist-led prescribing teaching [9,10]. Other proposed mechanisms include reduced learner anxiety and a cognitive load calibrated to the tutor's own recent experience, which may help explain why near-peer formats generalize across specialties [18]. RadPulse's emphasis on live BNF navigation under timed conditions, a gap participants specifically attributed to their own medical schools, is consistent with this account: the value reported was not additional factual content but demonstration of how to retrieve and apply information under the conditions the exam itself imposes.

The reported pass rate of 99.0% warrants cautious interpretation rather than straightforward comparison with the national benchmark. Only 19.0% of registrants returned an outcome, and students who volunteer this information are plausibly more engaged and confident than nonresponders; nothing in the design of the outcomes form would have stopped a student who performed poorly from simply not responding. The comparator itself is imperfect: it reflects cohorts sitting the PSA up to 2023, predating the January 2025 sitting attended by RadPulse students, so any apparent consistency with the national figure should be read as reassuring rather than confirmatory. A comparable pattern appears in a virtual transition-to-internship boot camp for final-year students, where 94% of participants scored above a comprehension threshold on an objective postcourse assessment alongside a significant rise in self-reported confidence [19]; as in that study, the self-report and objective components here were not designed to separate a genuine course effect from the characteristics of students who chose to take part. Taken together, this pass rate is best treated as an optimistic estimate among engaged respondents, not a population-level statistic.

The same self-selection concern limits the registration-Session 8 preparedness comparison, since these were anonymous, unlinked samples and not necessarily the same individuals responding at both timepoints; no inferential test was therefore applied. A further, more general limitation applies to how preparedness itself was measured: single-item self-report scales of this kind are known to be susceptible to social-desirability and recall effects, and their apparent simplicity should not be mistaken for validity [20].

Exploratory associations were weak throughout. Attendance was not associated with score among the students reporting both, and the association between a school's baseline provision and its students' subsequent uptake of RadPulse, while directionally positive, fell short of statistical significance given only 13 school-level units; neither should be overinterpreted. These null and marginal findings also expose a structural difficulty in applying Kirkpatrick's framework to an evaluation of this kind. The framework was designed for simpler, short-term instructional settings, and its higher levels do not map cleanly onto an examination outcome measured months later in a self-selected, unlinked sample, a mismatch that critical appraisals of Kirkpatrick's use in medical education have raised more generally [21]. Read together, RadPulse demonstrates that a free, near-peer course can achieve substantial national reach and high satisfaction with minimal infrastructure; whether it changes examination performance beyond what self-selected, motivated students would have achieved regardless remains an open question that this design cannot answer. Addressing the gap identified at the outset would require the comparative, linked-outcome design described above, rather than an evaluation of this kind.

Limitations

This evaluation has substantial limitations. Self-selection at every stage means registrants, session-feedback respondents, and outcomes-form respondents are unlikely to represent all UK final-year students, or even all RadPulse registrants. No linked or pseudonymized identifiers were used across data collection points; this protected anonymity but precluded individual-level pre/post comparison. Self-rated preparedness was measured with an unvalidated single-item scale. The 19.0% outcomes response rate is low, and nonresponse is plausibly related to performance, biasing the reported pass rate upward. This limitation applies specifically to the PSA pass-rate outcome, whereas the session-feedback and preparedness measures reported under Kirkpatrick Levels 1 and 2 drew on much larger, though still self-selected, samples. Percentage scores were optional, self-reported, and disclosed by only a further subset of an already self-selected sample, with no independent verification. There was no comparison group, so the observed associations cannot be attributed to the course itself but rather to the characteristics of students who choose to register for extracurricular prescribing teaching. Teaching content relied on tutors' recent PSA experience without independent senior clinical or pharmacological review. Qualitative coding was performed by two readers with disagreements resolved by discussion, but interrater agreement was not formally quantified, and thematic saturation was not assessed. Finally, evaluation was limited to Kirkpatrick Levels 1, 2, and 4, with Level 4 operationalized as examination outcome rather than organizational or patient-level impact; Level 3 (behavior) could not be assessed, as participants had not yet begun independent prescribing, so this study cannot speak to whether RadPulse changed prescribing practice or patient safety.

Implications for future research

A stronger evaluation would use consented, linked identifiers rather than anonymous forms, objective blueprint-matched pre- and posttesting rather than self-rated preparedness, automatic attendance capture, and complete outcome linkage for the full registrant cohort. A concurrent comparator, cluster design, or stepped-wedge rollout could test whether this association reflects a course effect or self-selection. Follow-up into foundation training would be needed to examine whether any benefit extends to prescribing decisions, error rates, or patient safety, none of which this study can address.

## Conclusions

A free, national, near-peer online PSA preparation program run by junior doctors reached 515 final-year students across 13 UK medical schools without institutional support or funding. Learner satisfaction was consistently high, self-rated preparedness was higher in later sessions than at registration in these unlinked samples, and most students who reported an outcome had passed the PSA. These data establish reach, feasibility, and acceptability; they do not establish a causal effect on examination performance, prescribing competence, or patient safety. The model appears low-cost and may offer a scalable model that other institutions could consider adapting, but a comparative, linked-outcome design would be needed before any claim of effectiveness could be made.

## Appendices

The following appendices reproduce the wording and response formats of the data-collection instruments used in this study, referred to in the Materials and Methods section. All instruments were administered online (Google Forms, Google LLC, Mountain View, CA). Fields collecting only email address, medical school, and year of study (used for de-duplication and school-level analysis, not as outcome measures) are omitted from the item lists below where they were common to every instrument.

Appendix A: registration form

Completed by students at the time of registration, prior to attending Session 1.

1. Email address (required)

2. Name (required)

3. Medical school (required)

4. Year of medical school (required)

5. How well prepared do you feel for the PSA? (5-point scale: 1 = Not Well Prepared to 5 = Very Well Prepared; required)

6. Do you feel as though your medical school has provided you with adequate resources for the PSA? (5-point scale: 1 = Not Much to 5 = Very Well)

7. Is there anything specific you would like us to cover in our PSA sessions? (free text; required)

Appendix B: session feedback forms (sessions 1-9)

Administered immediately after each of the nine sessions. Every form began with required fields for email address, medical school, and year of study, followed by the session-specific items below.

Sessions 1, 2, 3, and 5 (Single-Topic Sessions)

1. The content was presented at an appropriate level: (5-point Likert scale: Strongly disagree - Strongly agree)

2. The aims and objectives of the session were clearly defined: (5-point Likert scale: Strongly disagree - Strongly agree)

3. The structure and flow of the presentation were easy to follow: (5-point Likert scale: Strongly disagree - Strongly agree)

4. What elements of the session did you find particularly effective? (free text)

5. What changes would you recommend for future sessions? (free text)

Session 4: Prescription Review and Planning Management

1. Prescription Review: The content was presented at an appropriate level: (5-point Likert scale: Strongly disagree - Strongly agree)

2. Planning Management: The content was presented at an appropriate level: (5-point Likert scale: Strongly disagree - Strongly agree)

3. Prescription Review: The aims and objectives of the session were clearly defined: (5-point Likert scale: Strongly disagree - Strongly agree)

4. Planning Management: The structure and flow of the presentation were easy to follow: (5-point Likert scale: Strongly disagree - Strongly agree)

5. What elements of the session did you find particularly effective? (free text)

6. What changes would you recommend for future sessions? (free text)

Session 6: Adverse Drug Reactions and Drug Monitoring

1. Adverse Drug Reactions: The content was presented at an appropriate level: (5-point Likert scale: Strongly disagree - Strongly agree)

2. Drug Monitoring: The content was presented at an appropriate level: (5-point Likert scale: Strongly disagree - Strongly agree)

3. Adverse Drug Reactions: The aims and objectives of the session were clearly defined: (5-point Likert scale: Strongly disagree - Strongly agree)

4. Drug Monitoring: The structure and flow of the presentation were easy to follow: (5-point Likert scale: Strongly disagree - Strongly agree)

5. What elements of the session did you find particularly effective? (free text)

6. What changes would you recommend for future sessions? (free text)

Session 7: Data Interpretation

1. The content was presented at an appropriate level: (5-point Likert scale: Strongly disagree - Strongly agree)

2. The aims and objectives of the session were clearly defined: (5-point Likert scale: Strongly disagree - Strongly agree)

3. What elements of the session did you find particularly effective? (free text)

4. What changes would you recommend for future sessions? (free text)

5. When would you like us to do the Q&A session (before or after Christmas)? (scheduling logistics item; not an outcome measure)

Session 8: High Yield and Q&A

1. The content was presented at an appropriate level: (5-point Likert scale: Strongly disagree - Strongly agree)

2. The aims and objectives of the session were clearly defined: (5-point Likert scale: Strongly disagree - Strongly agree)

3. What elements of the session did you find particularly effective? (free text)

4. What changes would you recommend for future sessions? (free text)

5. How prepared do you feel now for the PSA exam after the course? (5-point scale: 1 = Not Very Prepared to 5 = Very Prepared) - source of the post-course preparedness measure reported in the Results.

Session 9: Mock Exam and Overall Course Feedback

1. The content was presented at an appropriate level: (5-point Likert scale: Strongly disagree - Strongly agree)

2. The aims and objectives of the session were clearly defined: (5-point Likert scale: Strongly disagree - Strongly agree)

3. What elements of the session and the course did you find particularly useful? (free text)

4. What changes would you recommend for future sessions? (free text)

5. How much has your confidence to sit the PSA improved with this course? (5-point scale: 1 = Not Very Prepared to 5 = Very Prepared) - source of the Session 9 confidence measure reported in the Results.

6. How useful did you find the course? (5-point scale: 1 = Not Very Useful to 5 = Very Useful) - source of the Session 9 usefulness measure reported in the Results.

Appendix C: post-examination outcomes form

A voluntary, anonymous form circulated after the January 29, 2025, national PSA sitting. Source of the pass-rate and disclosed-score measures reported in the Results.

1. Medical school (required)

2. Number of sessions of our course attended (0-9; required)

3. Did you pass the PSA? (Yes / No; required) - source of the reported PSA pass rate.

4. If you feel comfortable to share anonymously, what % did you achieve (to the nearest whole number)? (free text, optional) - source of the disclosed mean PSA score.
